# Gene expression profiling on effect of aspirin on osteogenic differentiation of periodontal ligament stem cells

**DOI:** 10.1038/s41405-021-00090-5

**Published:** 2021-09-16

**Authors:** Fazliny Abd Rahman

**Affiliations:** Faculty of Dentistry, SEGi University, Kota Damansara, 47810 Petaling Jaya, Selangor Malaysia

**Keywords:** Dentistry, Dental conditions

## Abstract

Periodontal ligament (PDL) contains a unique population of mesenchymal stem cells (MSCs), also known as PDL stem cells (PDLSCs). The regenerative properties of PDLSCs hold great potential for its use in stem cells based therapy, particularly for periodontal or bone regeneration. The present study investigated the global gene expression profile in PDLSCs during osteogenic differentiation. MSCs from PDL were isolated from normal permanent human teeth (*n* = 3). Microarray analysis was used to study the effects of ASA (200, 500, and 1000 μM) on the gene expression profiles in PDLSCs during osteogenic differentiation. Microarray study revealed that ASA was able to modulate PDLSCs gene expression profile. At 200 µM, 315 genes were dysregulated genes (DE), involving 151 upregulated and 164 downregulated genes. At 500 µM, 794 genes were DE, involving of 364 upregulated and 430 downregulated genes. At 1000 µM, the number of DE genes increased to 2035, of which 735 were upregulated and 1300 were downregulated. Bioinformatics analyses of the gene expression data revealed that the majority of DE genes (for 500 and 1000 µM ASA treatment) are involved in osteogenic differentiation. The gene network analysis was carried out using Ingenuity Pathway Analysis (IPA) software, and this revealed that the number of gene groups involved in cell adhesion and extracellular matrix components were increased. This study indicated that ASA could enhance PDLSCs functions and provide evidence for the potential use of ASA with PDLSCs for regenerative dentistry applications, particularly in the areas of periodontal health and regeneration. Periodontal ligament stem cells (PDLSCs) Aspirin (ASA) Microarray Osteogenic

## Introduction

Mesenchymal stem cells (MSCs) are multipotent cells and they become a prominent subject in regenerative medicine because of their capability to self-renew and differentiate into trilineage differentiation. MSCs were first discovered by Friedenstein et al. in the rodent bone marrow, which they called bone marrow mesenchymal stem cells (BMMSCs) [1]. Since then, knowledge from the study of BMMSCs has been used as the “gold standard” in the approach to the study of stem cells [2]. However, the procedure for obtaining BMMSCs is invasive and the harvesting yield is low [3]. Thus, the isolation of MSCs from dental tissues as an alternative to BMMSCs provides an appealing alternative. The dental stem cells can be obtained with ease as they are readily accessible in the oral environment and can even be obtained from extracted teeth that are usually discarded. Periodontal ligament stem cells (PDLSCs) is one of the source of dental pulp stem cells [4]. Periodontal regeneration is a method of regenerative therapy to return the periodontal tissues (including gingiva, root cementum, alveolar bone, and the periodontal ligament) to their original healthy condition through restoration of form and function of lost structures [5]. Non-steroidal anti-inflammatory drugs (NSAID) are widely used as an analgesic agent in healthcare. It may be used in managing orthopedic patients pre/post-surgery, to address acute or chronic inflammation. However, such use of NSAIDs may have undesirable impact on stem cells function, particularly in periodontal or bone regeneration [6–10].

Aspirin (ASA) is renowned NSAID that has been used for decades. ASA has been reported to modulate a variety of conditions related to human disease, such as cardiovascular disease, periodontal health, cancer and diabetes [11, 12]. The impact of ASA on stem cells properties has been reported in a number of studies [13–15]. However, not many studies have examined the effects of ASA on MSCs or osteoblast precursor cells. The beneficial or adverse effects of ASA on the survival and function of MSCs, particularly in osteogenic differentiation needs to be further investigated. Our previous study showed that ASA is capable of enhancing the proliferation and osteogenic differentiation of PDLSCs grown in osteogenic media [16]. We further investigate the effect of ASA of PDLSCs grown in osteogenic media for continuation of our study. The present study examined the effect of ASA (200, 500, and 1000 μM) on PDLSCs osteogenic differentiation through microarray gene expression profiling. This study sought to assess the significance effect of ASA treatments at 200, 500, and 1000 μM on the osteogenic potential of PDLSCs. It employed microarray assays in studying the effect of ASA on the osteogenic potential of PDLSCs using the platform of Agilent SurePrint G3 Human GE v2 8×60K Microarrays.

The aim of this study is to analyze the effect of ASA on PDLSCs gene expression profiles during osteogenic differentiation through microarray analysis and bioinformatics analysis of gene expression data to gain insights on the mechanism/impact of ASA on PDLSCs osteogenic differentiation.

## Methodology

### Isolation and culturing human PDLSCs from periodontal ligament tissue

This study was approved by the Medical Ethics Committee, Faculty of Dentistry, University of Malaya [Medical Ethics Clearance Number: DF CO1107/0066 (L)] and with the informed consent of the patients. The PDLSCs were isolated from normal and vital tooth. The donors were aged between 18 and 35 years old (*n* = 3) and the teeth were indicated for extraction for orthodontics treatment purposes. The PDLSCs were isolated by using standard protocols with some modifications [4]. The PDL tissues were scraped off the root surface using a sterilized scalpel and minced into small fragments prior to digestion in a solution of 1 mg/mL collagenase type I (Gibco, Grand Island, NY) for 30 min at 37 °C.

After neutralization with a 10% fetal bovine serum (FBS) (Gibco, Grand Island, NY), the cells were centrifuged and seeded in T75 culture flasks (BD Pharmingen, San Diego CA, USA) using a culture medium containing KO-DMEM, 10% FBS, 0.5% and 10,000 µg/mL of penicillin/streptomycin (Invitrogen), 1% 1× Glutamax (Invitrogen), and incubated at 37 °C in the presence of 5% CO_2_. The medium was replaced every 3 days until the cells reach 70–80% confluency

### The effects of ASA on PDLSCs osteogenic potential

The PDLSCs were seeded in T75 culture flasks (BD Pharmingen) and maintained in 5% CO_2_ incubator at 37 °C for 24 h until they reached 70–80% confluency. Then, the PDLSCs (*n* = 4) were exposed to ASA [0 (control), 200, 500, 1000 μM] in osteogenic media containing 10% FBS, 1% l-alanyl-l-glutamine, 100 nM dexamethasone, 10 mmol/L β-glycerol phosphate and 0.2 mM of ascorbic acid for 21 days. The media was changed every 3 days. Total RNA was extracted at day 21, using the RNAeasy Mini kit (Qiagen).

### RNA isolation

The RNA concentration was quantified using a Nanodrop spectrophotometer ND-2000 (Thermo Scientific Inc), and the RNA integrity number was determined using Agilent Bioanalyzer 2100 (Agilent Technologies).

### Microarray analysis

The microarray assay was carried out using Agilent SurePrint G3 Human GE v2 8x60K Microarrays (Agilent Technologies, catalog number G4851B) according to the manufacturer’s protocol. One hundred nanograms of total RNA was labeled with Low Input Quick Amp Labeling Kit, One color (Agilent Technologies) following the manufacturer’s instruction. In this step, 100 ng of total RNA was converted into double-stranded cDNA by priming with an oligo-dT primer containing the recognition site for T7 RNA polymerase. In vitro transcription with T7 RNA polymerase was used to produce cyanine 3-CTP labeled cRNA.

Six hundred nanograms of labeled cRNA was hybridized onto Agilent SurePrint G3 Human GE 8X60K Microarray for 17 h at 65 °C in hybridization oven (10 rpm). After hybridization, the microarray slide was washed in gene expression wash buffer 1 for 1 min at room temperature and another minute in gene expression wash buffer 2 at 37 °C before scanning the chip on Agilent High Resolution Microarray Scanner (C-model). Raw signal data were extracted from TIFF image with Agilent Feature Extraction Software (V10.7.1.1). Repeated measure test was used for statistical analysis. Genes up or downregulated (*p* < 0.05) by two-fold change (FC) were selected for further analysis.

### Gene ontology (GO) and functional enrichment analysis

The PANTHER (protein analysis through evolutionary relationships) classification system provides intuitive visualizations for GO analysis output and this was used to classify the differentially expressed genes to facilitate data analysis. The functional enrichment analyses were performed using DAVID functional annotation clustering tool [17] to ascertain the effect of ASA treatment at 200, 500, and 1000 μM on the gene expression profile. The categories were filtered based on the enrichment score with the lowest *p* values. The threshold value of the enrichment score was set at 1.6. DAVID generates an enrichment score for a group of genes indicating the associations of annotation term members in a given experiment.

### Functional annotation and pathway analysis

Pathway enrichment analysis was performed using Qiagen’s Ingenuity Pathways Analysis (IPA, Ingenuity Systems Inc.). The software determines the significance (*p* value) of a particular pathway being represented in the list of differentially expressed genes by Fisher’s exact. IPA is also able to show the canonical pathway participated by any of the dysregulated genes, the gene functions, and the potential gene network interactions.

A dataset with gene identifiers and corresponding FC values were entered into IPA software. These molecules were overlaid onto a global molecular network (contained in the Ingenuity Knowledge Base) during the analyses. Default settings were used to perform the analyses. The functional analysis in IPA allows determination of the biological functions and diseases associated with each network. Since the data composition of Ingenuity Knowledge Base can change over time, the results of the IPA analyses performed at different times may differ in the details uncovered.

### Validation microarray

#### Real-time PCR (qPCR)

The microarray results were validated by Real-time PCR (qRT-PCR). A number of genes that were detected to be differentially expressed were selected and they are as listed in Table 1. The validation assay was carried out using the corresponding TaqMan^®^ Gene Expression Assay ID (Thermo Fisher Scientific) reagents set. All determinations were normalized using GAPDH as the reference gene.

**Table 1:** List of TaqMan primers for microarray validation.

No.	Taqman primers	Assay ID
1.	Fibroblast growth factor 1 (FGF1)	Hs01092738_m1
2.	Fibroblast growth factor 5 (FGF5)	Hs03676587_s1
3.	Fibroblast growth factor receptor-like 1 (FGFRL1)	Hs00222484_m1
4.	Integrin, alpha 5 (fibronectin receptor, alpha polypeptide) (ITGA5)	Hs01547673_m1
5.	Fibronectin 1 (FN1)	Hs01549976_m1
6.	BMP binding endothelial regulator (BMPER)	Hs00403062_m1
7.	Bone morphogenetic protein 4 (BMP4)	Hs03676628_s1

### Western blotting

Protein was extracted using RIPA buffer with protease inhibitors. Fifty micrograms of the samples was heated in 5× loading dye at 95 °C for 15 min and then loaded onto a 10% SDS–PAGE gel. After electrophoresis, the proteins were transferred to an Immobilon^®^ PVDF membrane (Millipore, Bedford, MA) which was blocked with 5% bovine serum albumin and incubated overnight with the primary antibody. The membrane was washed with PBST and incubated with a secondary antibody for 1 h. After the wash cycle, the protein bands were detected with Luminata Crescendo Western HRP substrate detection reagent (Millipore).

### Statistical analysis

The results are presented as means ± standard deviations of at least three replicates (*n* = 3). Two-way ANOVA with Bonferroni post-test was performed using software (Graphpad Software, San Diego CA) and *p* values of <0.05 were considered significant. The PCR array data was analyzed at Qiagen’s data analysis center (http://pcrdataanalysis.sabiosciences.com/pcr/arrayanalysis.php). The *p* values were calculated based on a student’s *t*-test of the triplicate 2^ (-Delta Ct) (2 FC) values for each gene in the control and treatment groups with *p* values < 0.05 being considered significant.

## Results

### ASA modulation of gene expression profile during PDLSCs osteogenic differentiation

Data processing, normalization, and error modeling were performed using Genespring GX (Agilent Technologies). In the present study, the Agilent microarray being used is contained eight arrays/slide, of which 60,000 features are found in each of the arrays. Each feature/gene identifier could be mapped to the relevant/corresponding gene. The highest expression value was selected in the case of genes with multi-identifiers.

**Fig. 1:**
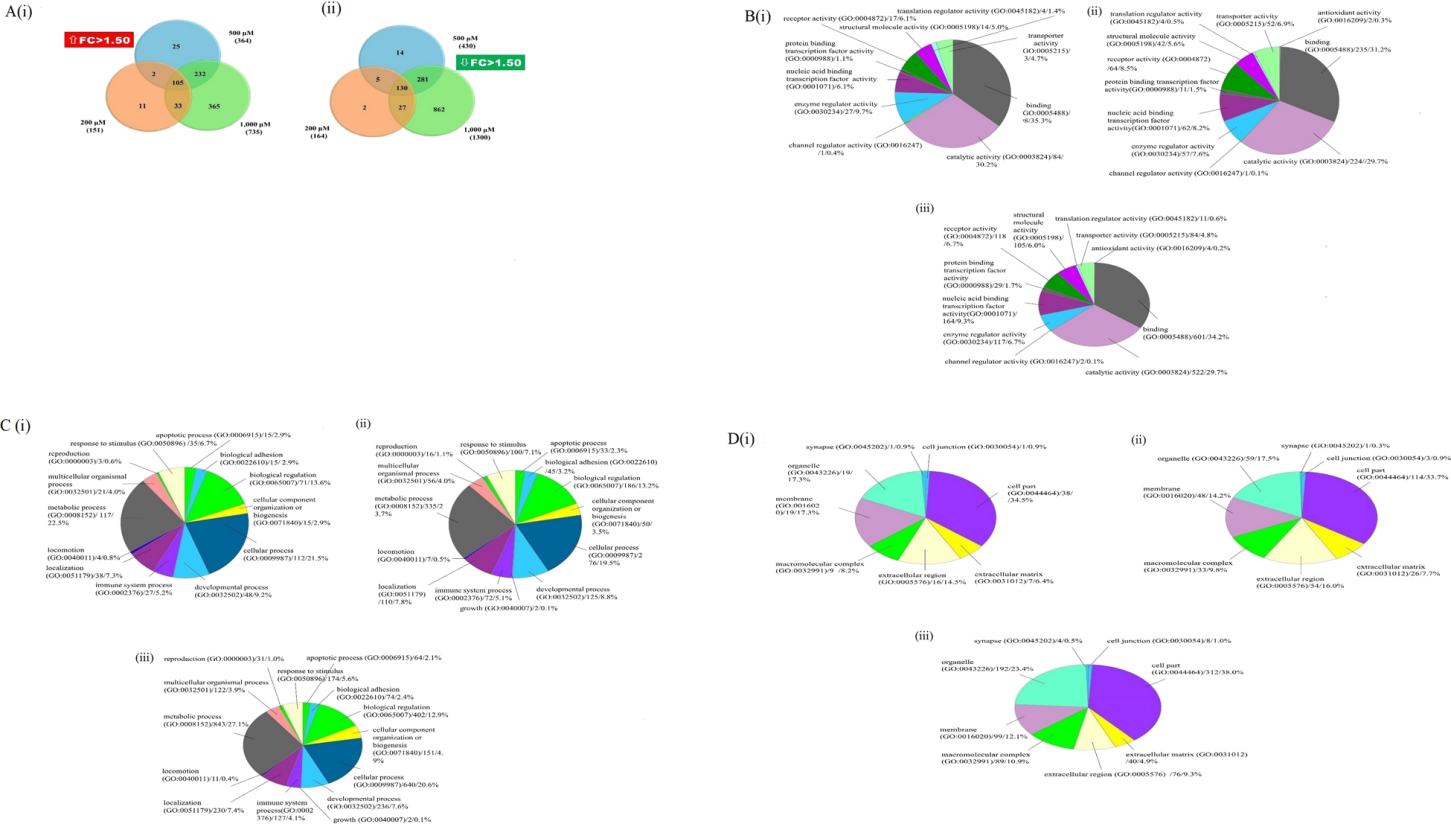
Venn diagram and Functional analyses of differentially expressed genes of ASA treatments. **A** Venn diagram representing the number of DEGs that are shared among the 200, 500, and 1000 μM ASA-treated samples. (FC 1.5: Fold Change Upregulated); B(ii)FC 1.5: Fold Change Downregulated; **B** Functional analyses of differentially expressed genes by PANTHER. Representative GO terms of Biological Processes (BP) at **B**(i) 200 μM, **B**(ii) 500 μM and **B**(iii) 1000 μM of ASA treatments. C; Functional analyses of differentially expressed genes by PANTHER. Representative GO terms of Molecular Functions (MFs) at **C**(i) 200 μM, **C**(ii) 500 μM and **C**(iii) 1000 μM of ASA treatments. Functional analyses of differentially expressed genes by PANTHER. Representative GO terms of cellular components (CCs) at **D** (i) 200 μM, **D**(ii) 500 μM and **D**(iii) 1000 μM of ASA treatments.

Using an FC of 1.5 as a cut-off threshold value (*p* < 0.05), 3144 were found to be DEGs (Fig. 1A). At 200 μM ASA treatment, 315 DEGs were noted, including 151 upregulated (Fig. 1A(i)) and 164 downregulated genes (Fig. 1A(ii)). At 500 μM, 794 DEGs were identified, including 364 upregulated (Fig. 1A(i)) and 430 downregulated genes (Fig. 1A(ii)). In contrast, at 1000 M, 2035 DEGs were noted, which included 1250 upregulated and 1894 downregulated genes.

### Gene ontology (GO) for ASA treatment

Genes ontology (GO) analysis was enriched by using PANTHER families [18]. GO analysis was used to infer functions shared among related genes. Gene family phylogeny was used to incorporate the variant experimentally derived GO functional annotations across related genes. It classifies the 1344 genes (FC ≥ 1.5, *p* < 0.05) according to biological process (BP), molecular function (MF), and cellular component (CC). Figure 1B–D shows BP, MF, and CC for 200, 500, and 1000 μM of ASA treatments, respectively.

The distribution of the genes conferring BP at 200 μM of ASA treatments (Fig. 1B) is as follows: metabolic process (117 genes, 22.5%), cellular process (112 genes, 21.5%), biological regulation (71 genes, 13.6%), localization (38 genes, 7.3%), developmental process (48 genes, 9.2%), response to stimulus (35 genes, 6.7%), immune system process (27 genes, 5.2%), multicellular organismal process (21 genes, 4.0%), CC organization or biogenesis (15 genes, 2.9%), apoptotic process (15 genes, 2.9%), locomotion (4 genes, 0.8%), biological adhesion (15 genes, 2.9%), and reproduction (3 genes, 0.6%),

The distribution of the genes conferring BPs at 500 μM of ASA treatments (Fig. 1C) is as follows: metabolic process (335 genes, 23.7%), cellular process (276 genes, 19.5%), biological regulation (186 genes, 13.2%), developmental process (125 genes, 8.8%), localization (110 genes, 7.8%), response to stimulus (100 genes, 7.1%), immune system process (72 genes, 5.1%), multicellular organismal process (56 genes, 4.0%), CC organization or biogenesis (50 genes, 3.5%), a biological adhesion (45 genes, 3.2%), apoptotic process (33 genes, 2.3%), reproduction (16 genes, 1.1%), locomotion (7 genes, 0.5%) and growth (2 genes, 0.1%).

The distribution of the genes conferring BPs at 1000 μM of ASA treatments (Fig. 1D) is as follows: metabolic process (843 genes, 27.1%), cellular process (640 genes, 20.6%), biological regulation (402 genes, 12.9%), developmental process (236 genes, 7.6%), localization (230 genes, 7.4%), response to stimulus (174 genes, 5.6%), CC organization or biogenesis (151 genes, 4.9%), immune system process (127 genes, 4.1%), multicellular organismal process (122 genes, 3.9%), biological adhesion (74 genes, 2.4%), apoptotic process (64 genes, 2.1%), reproduction (31 genes, 1.0%), locomotion (11 genes, 0.4%) and growth (2 genes, 0.1%).

The genes for MFs at 200 μM of ASA treatments (Fig. 1B) were for binding (98 genes, 35.3%), catalytic activity (84 genes, 30.2%), enzyme regulator activity (27 genes, 9.7%), receptor activity (17 genes, 6.1%), nucleic acid binding transcription factor activity (17 genes, 6.1%), structural molecule activity (14 genes, 5.0%), transporter activity (13 genes, 4.7%), translation regulator activity (4 genes, 1.4%), protein binding transcription factor activity (3 genes, 1.1%) and channel regulator activity (1 gene, 0.4%).

The genes for MFs at 500 μM of ASA treatments (Fig. 1C) were binding (235 genes, 31.2%), catalytic activity (224 genes, 29.7%), receptor activity (64 genes, 8.5%), nucleic acid binding transcription factor activity (62 genes, 8.2%), enzyme regulator activity (57 genes, 7.6%), transporter activity (52 genes, 6.9%), structural molecule activity (42 genes, 5.6%), protein binding transcription factor activity (11 genes, 1.5%), translation regulator activity (4 genes, 0.5%), channel regulator activity (1 gene, 0.1%) and antioxidant activity (2 genes, 0.3%).

The genes for MFs at 1000 μM of ASA treatments (Fig. 1D) were binding (601genes, 34.2%), catalytic activity (522 genes, 29.7%), nucleic acid binding transcription factor activity (164 genes, 9.3%), receptor activity (118 genes, 6.7%), enzyme regulator activity (117 genes, 6.7%), structural molecule activity (105 genes, 6.0%), transporter activity (84 genes, 4.8%), protein binding transcription factor activity (29 genes, 1.7%), translation regulator activity (11 genes, 0.6%), antioxidant activity (4 genes, 0.2%) and channel regulator activity (2 genes, 0.1%).

The genes for CC at 200 μM of ASA treatments (Fig. 1B) were cell part (38 genes, 34.5%), organelle (19 genes, 17.3%), membrane (19 genes, 17.3%), extracellular region (16 genes, 14.5%), macromolecular complex (9 genes, 8.2%), extracellular matrix (ECM, 7 genes, 6.4%), synapse (1 gene, 0.9%) and cell junction (1 gene, 0.9%). The genes for CCs at 500 μM of ASA treatments (Fig. 1C) were cell part (114 genes, 33.7%), organelle (59 genes, 17.5%), extracellular region (54 genes, 16.0%), membrane (48 genes, 14.2%), macromolecular complex (33 genes, 9.8%), ECM (26 genes, 7.7%), cell junction (3 genes, 0.9%) and synapse (1 gene, 0.3%). The genes for CCs at 1000 μM of ASA treatments (Fig. 1D) were cell part (312 genes, 38.0%), organelle (192 genes, 23.4%), membrane (99 genes, 12.1%), macromolecular complex (89 genes, 10.9%) and extracellular region (76 genes, 9.3%), ECM (40 genes,4.9%), cell junction (8 genes, 1.0%) and synapse (4 genes, 0.5%).

### ASA treatment and enrichment of canonical pathway

**Fig. 2:**
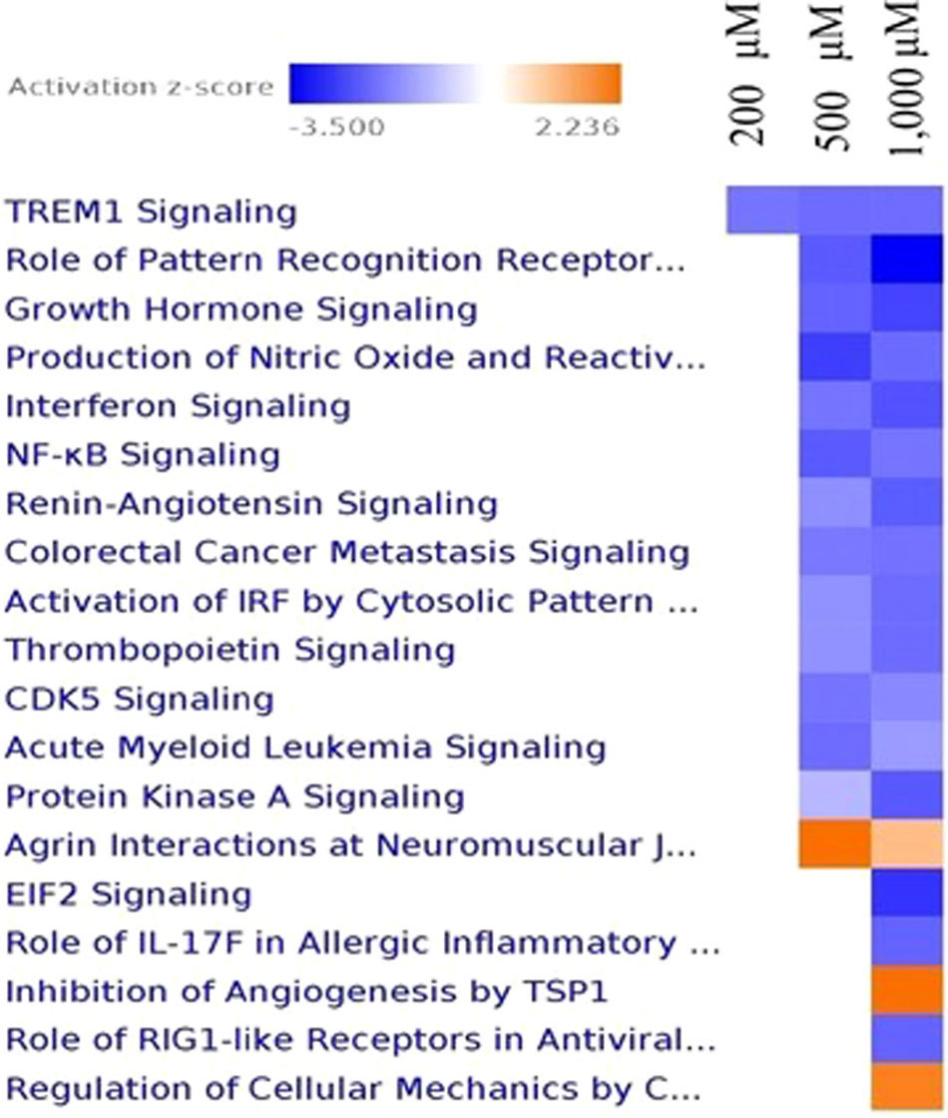
Heat map representing the relevant canonical pathways of ASA treatments at 200, 500, and 1000 μM. Most activated or inhibited canonical pathways as estimated by the *z*-score (±≥ 2) for each comparison from ingenuity Pathway analysis (IPA). Blue denotes predicted activation and orange predicted inhibition.

Figure 2 depicts the most enriched canonical pathways at 200, 500, and 1000 μM upon ASA treatments, arranged according to their *z*-scores. They can be seen to have similar patterns/consistent activation or inhibition at all concentrations of ASA treatments. The pathways which were predicted to be significantly activated at 500 μM of ASA treatments included only Agrin interactions at neuromuscular junctions (*z*-score: 2.236068) (Table 2). Meanwhile at 1000 μM of ASA treatments the inhibition of angiogenesis by TSP1 (z-score: +2.236068) (Fig. 2) and regulation of cellular mechanics by calpain protease (z-score: +2.236068). The other canonical pathways were predicted to be inhibited at 500 and 1000 of ASA treatments (Fig. 2). Only the TREM1 signaling pathway was predicted to be significantly inhibited at all concentrations of ASA treatments (200, 500, and 1000 μM).

**Table 2:** Relevant upstream transcription regulators at 200, 500, and 1000 μM of ASA treatments.

Canonical pathway	200 μM	500 μM	1000 μM
TREM1 signaling	−2.0	−2.1213	−2.1106
Role of pattern recognition receptors in recognition of bacteria and viruses	N/A	−2.3333	−3.5000
Growth hormone signaling	N/A	−2.2361	−2.6458
Production of nitric oxide and reactive oxygen species in macrophages	N/A	−2.7136	−2.13200
Interferon signaling	N/A	−2.000	−2.44949
NF-κB signaling	N/A	−2.333333	−2.0000
Renin-angiotensin signaling	N/A	−1.6667	−2.3238
Colorectal cancer metastasis signaling	N/A	−1.9415	−2.0412
Activation of IRF by cytosolic pattern recognition receptors	N/A	−1.6330	−2.12132
Thrombopoietin signaling	N/A	−1.6330	−2.1213
CDK5 signaling	0.0	−2.000	−1.73205
Acute myeloid leukemia signaling	N/A	−2.1213	−1.5076
Protein kinase A signaling	N/A	−1.14708	−2.3591
Agrin interactions at neuromuscular junction	0.0	2.23607	1.1339
EIF2 signaling	0.0	N/A	−2.8402
Role of IL-17F in allergic inflammatory airway diseases	N/A	N/A	−2.2361
Inhibition of angiogenesis by TSP1	N/A	N/A	2.23607
Role of RIG1-like receptors in antiviral innate immunity	0.0	N/A	−2.2360
Regulation of cellular mechanics by calpain protease	0.0	N/A	2.0000

Most activated or inhibited upstream transcription regulators as estimated by the *z*-score for each comparison from Ingenuity Pathway Analysis.

*N/A* not available.

### Molecular and cellular functions

The microarray data were further analyzed using IPA to identify categories of molecular and cellular functions (MCFs) that were highly regulated for 200, 500, and 1000 μM of ASA treatments during PDLSCs osteogenic differentiation. Genes with FC of >1.5 were selected and processed for the analyses. Each of the significantly regulated MCFs that was identified at 200, 500, and 1000 μM of ASA treatments are associated with categories of processes, each with their own z-score, to indicate the relevant category state of activation, if any. Categories having z-score ≥ 2 are predicted by IPA to be in activated state, while those with *z*-score of ≤ − 2, were deemed not activated

The top five MCFs that were significantly regulated (p < 0.05) at 200 μM of ASA treatment were: cellular movement, cell to cell signaling, cellular compromise, cellular growth and proliferation, and lipid metabolism (Table 3).

**Table 3:** Top five MCFs predicted by IPA for 200 μM of ASA treatment (p < 0.05).

Molecular and cellular functions	Number of genes involved	*p* value
Cellular movement	18	2.45E-02-9.81E-06
Cell to cell signaling and interaction	21	2.70E-02-1.53E-05
Cellular compromise	8	2.70E-02-1.53E-05
Cellular growth and proliferation	56	2.35E-02-2.16E-05
Lipid metabolism	11	2.19E-02-2.99E-05

The top five MCFs that were significantly regulated (p < 0.05) at 500 μM of ASA treatment were: cellular growth and proliferation, cellular development, cellular movement, cellular function and maintenance and cell death and survival (Table 4).

**Table 4:** Top five MCFs as predicted by IPA for 500 μM ASA treatment (*p* < 0.05).

Molecular and cellular functions	Number of genes involved	*p* value
Cellular growth and proliferation	164	1.12E-02-2.54-09
Cellular development	123	1.12E-02-2.81E-07
Cellular movement	95	1.09 E-02-E-05
Cellular function and maintenance	34	E-02-E-05
Cell death and survival	124	2.06E-02-4.43E-05

The top five MCFs that were significantly regulated (*p* < 0.05) at 1000 μM of ASA treatment were: hematological system development and function, hematopoiesis, tissue development, cardiovascular system development and function and organismal development (Table 5).

**Table 5:** Top five MCFs predicted by Ingenuity Pathway Analysis (IPA) that were corresponding to 1000 μM treatment (*p* < 0.05).

Molecular and cellular functions	Number of genes involved	*p* value
Gene expression	300	1.52E-02-1.37E-14
Cell death and survival,	273	1.68E-02-1.24E-08
Cellular growth and proliferation	341	1.48E-02-3.77E-08
Post-translational modification	61	8.89E-02-9.10E-06
Protein synthesis	123	7.68E-02-9.10E-06

### Heat map of upstream regulators

**Fig. 3:**
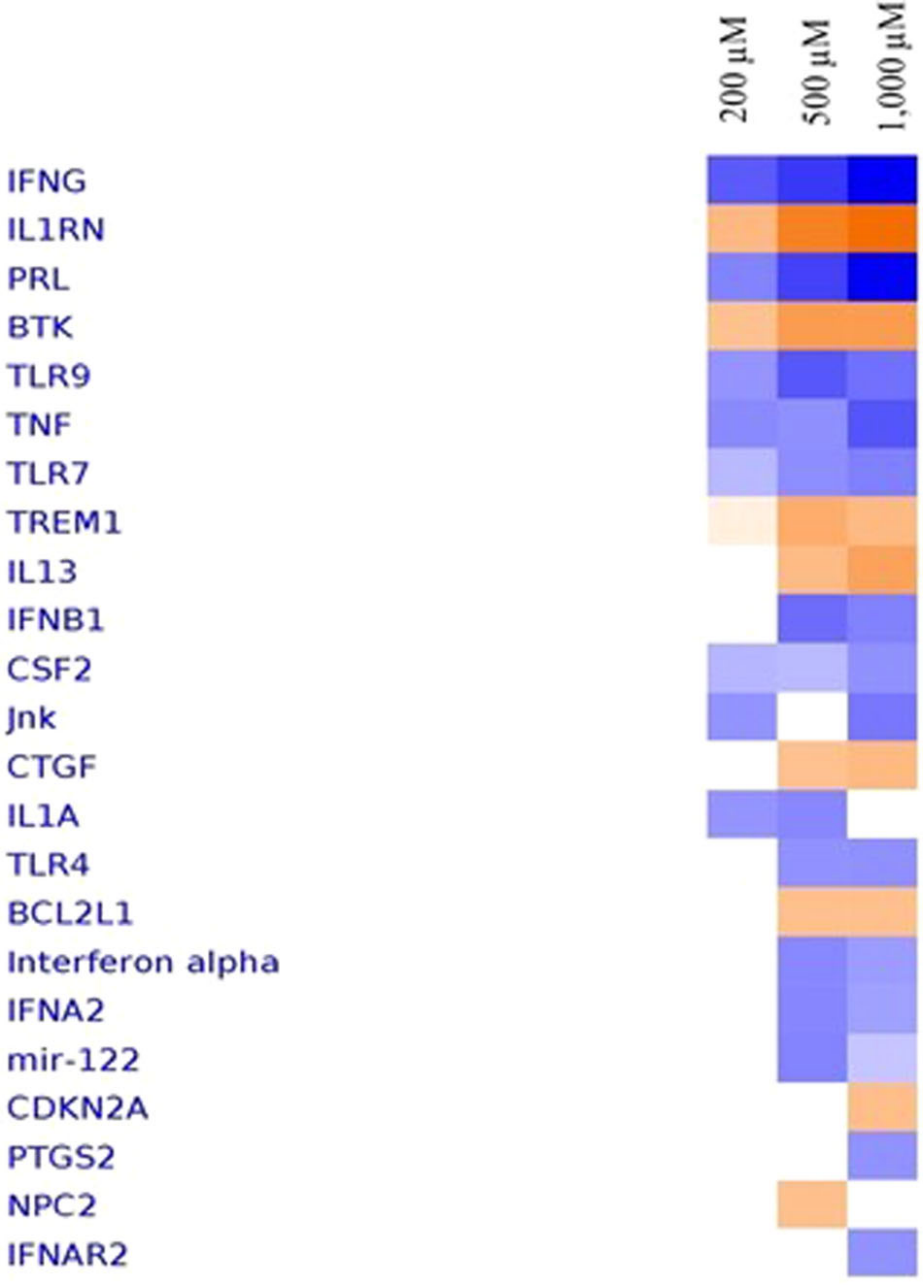
Relevant upstream transcription regulators at 200, 500, and 1000 μM of ASA treatments. Most activated or inhibited upstream transcription regulators as estimated by the **z**-score for each comparison from Ingenuity Pathway Analysis. Orange denotes predicted activation and blue predicted inhibition.

Upstream analysis provided a list of inferred upstream regulators based upon measured FCs for each concentration of ASA treatments. The objective of the Upstream regulator analysis (URA) is to identify a putative cascade of upstream transcriptional regulators (TRs) that interpret the observed gene expression changes in a user’s dataset. Figure 3 depicts the most enriched upstream regulators at 200, 500, and 1000 μM upon ASA treatments according to the significant *z*-scores.

They can be seen to have similar patterns/consistent activation or inhibition at all concentrations of ASA treatments. Few upstream regulators were shown to activate at all concentrations of ASA treatments including IL1RN and BTK. Meanwhile, few upstream regulators were shown to activate at 500 and 1000 μM of ASA treatments including IL1RN, BTK, TREM1, IL13, and BCL2L1. The only upstream regulator seen to activate at 1000 μM of ASA treatments is the CDKN2A.

### Validation of microarray data by qRT-PCR, immunofluorescence, and western blotting analyses

**Fig. 4:**
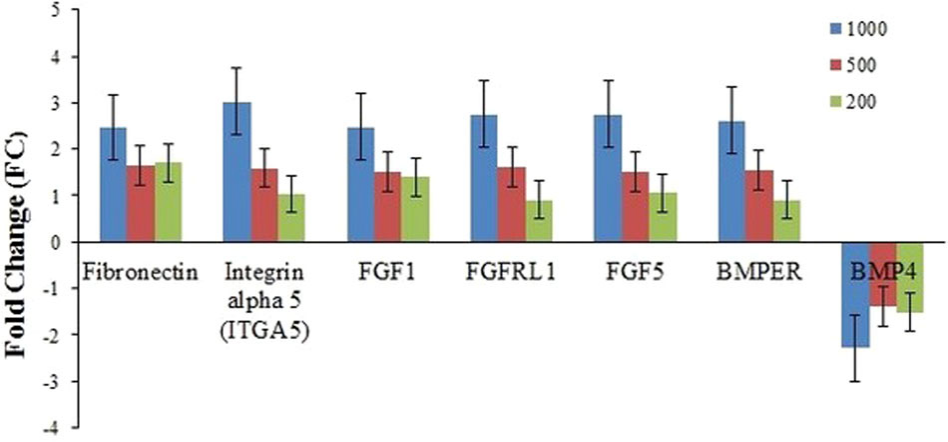
RT-qPCR analyses on effect of ASA in osteogenic differentiation of PDLSCs. Real-time RT-PCR analyses of FN1, Igα5, FGF1, FGF5, FGFRL1, BMP4, and BMPER on effect of ASA at 1000 μM in osteogenic differentiation of PDLSCs (*n* = 3);

Quantitative real time RT-PCR was done on seven differentially DE genes to validate the microarray data. Seven genes selected for validation were associated with osteogenesis differentiation. Our observations hypothesized that ASA promotes osteogenesis by targeting the FGF/FGFRL pathway and modulates fibronectin and integrin interaction. The genes include (Fibronectin 1) FN1, (integrin α5) Igα5, fibroblast growth factor-1 (FGF1), fibroblast growth factor-5 (FGF5), fibroblast growth factor receptor-like 1 (FGFRL1), bone morphogenic-4 (BMP4), and BMP binding endothelial regulator (BMPER). The result showed the genes significantly different expression rates between ASA-treated cells and untreated cells (Fig. 4). The qRT-PCR results were generally in agreement with the data obtained from microarray analyze.

**Fig. 5:**
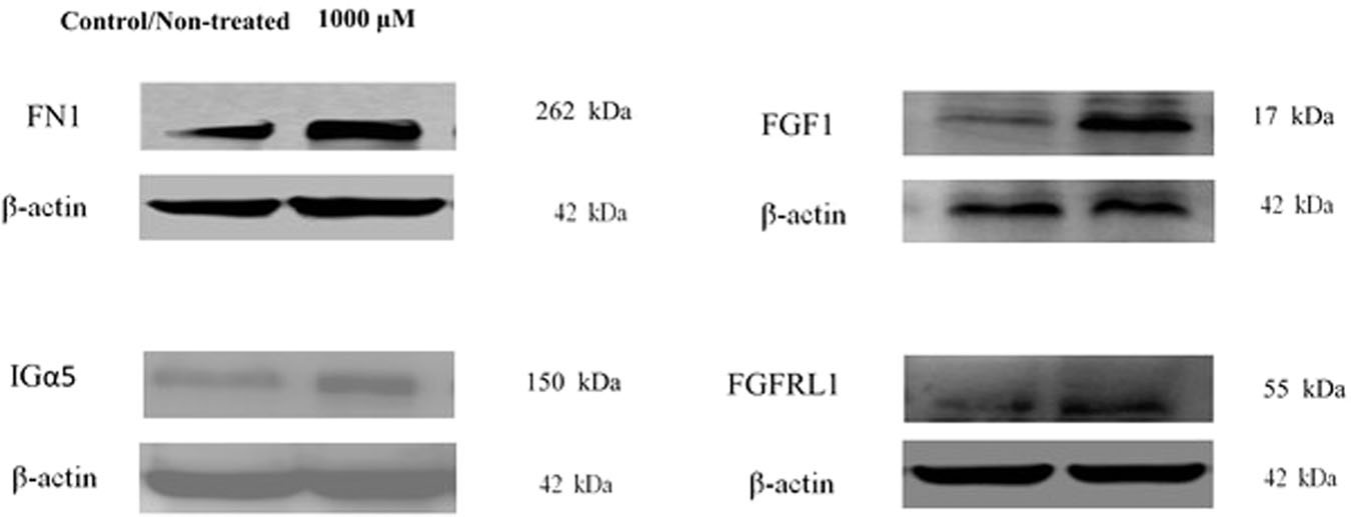
Western blot analyses. FN1, FGF1, FGFRL1, and Itgα5 proteins for control (non-treated) and 1000 μM of ASA treatments in osteogenic differentiation of PDLSCs (*n* = 3).

Figure 5 shows representative WB assay results for FN1, Itgα5, FGF1, and FGFRL1 genes for cells treated with 1000 μM ASA. The protein was expressed and the results were generally in agreement with the data obtained from microarray analyze.

## Discussion

### ASA modulation of gene expression profile during osteogenic differentiation

The PDLSCs isolated in the current study showed characteristics of MSCs as previously described [2, 4]. The PDLSCs were positive for CD73, CD90, and CD105 surface markers and negative for CD45, CD34, CD20, and CD14 hematopoietic markers. The range of ASA concentrations used in this study was within the in vivo therapeutic range of between 100 and 2000 µM as previously reported [16, 19, 20]. Based on the previous study, the low dose of ASA with the most significant effect at 1 mmol/L could increase osteogenic differentiation and osteogenic effect in osteoporotic rats [19]. They also observed that increased the secretion of ALP and promoted calcification when treated with low dose of ASA [19].

The present study also examined the effect of ASA (200, 500, and 1000 μM) on PDLSCs osteogenic differentiation through microarray gene expression profiling. Microarray technology is a powerful tool used in studying the level of expression of thousands of genes simultaneously. This study sought to assess the significance effect of ASA treatments at 200, 500, and 1000 μM on the osteogenic potential of PDLSCs. It employed microarray assays in studying the effect of ASA on the osteogenic potential of PDLSCs using the platform of Agilent SurePrint G3 Human GE v2 8x60K Microarrays.

The present study examined the effect of ASA (200, 500, and 1000 μM) on PDLSCs osteogenic differentiation through microarray gene expression profiling. The effect of PDLSCs on proliferation and osteogenic differentiation of PDLSCs on cellular levels are already discussed in our previous publications [16]. PDLSCs and osteogenic Microarray technology is a powerful tool used in studying the level of expression of thousands of genes simultaneously. This study sought to assess the significance effect of ASA treatments at 200, 500, and 1000 μM on the osteogenic potential of PDLSCs. It employed microarray assays in studying the effect of ASA on the osteogenic potential of PDLSCs using the platform of Agilent SurePrint G3 Human GE v2 8×60K Microarrays.

Using an FC of 1.5 as a cut-off threshold value (*P* < 0.05), 3144 genes were found to be DEGs. Among them, a total of 1765 genes (56.14%) were upregulated and 2488 genes (79.13%) were downregulated at all concentrations of ASA treatments. At 200 μM ASA treatment, 315 DEGs were noted, including 151 upregulated and 164 downregulated genes. At 500 μM, 794 DEGs were identified, including 364 upregulated and 430 downregulated genes. In contrast, at 1000 μM, 2035 DEGs were noted, which included 1250 upregulated and 1894 downregulated genes.

The data from microarray study was subjected to data mining, to uncover the plausible pathways activated or highly regulated by ASA in PDLSCs. The results indicated that ASA at 500 and 1000 μM were able to enhance osteogenic potential in PDLSCs. To significantly determine the importance of the biological function of genes, GO analysis was conducted using PANTHER and pathway enrichment analysis by DAVID.

### Gene ontology for ASA treatment

According to the PANTHER analysis, the BPs showed that both upregulated and downregulated genes in all ASA treatments (200, 500, and 1000 μM) had similar patterns which were highly enriched for metabolic and cellular processes, and for biological regulation. The regulation of metabolic pathways was shown to be involved in regulating the fate of stem cells. The MSCs metabolized to self-renew and differentiate in vitro. During the early stages of MSC differentiation, the fate of the new cells is redirected by downregulating the pluripotent specific genes and upregulating the terminal-specific genes and switching the subsets of metabolic enzymes [21].

MFs revealed that upregulated and downregulated genes in all ASA treatments showed similar patterns which were highly enriched for binding and catalytic and nucleic acid binding transcription factor activities. CCs showed that both types of genes in all ASA treatments showed similar patterns and were highly enriched in the cell part, organelle, and extracellular regions.

An interesting finding was that the highly abundant cell transcripts at 500 and 1000 μM ASA treatments were significantly enriched in functions related to protein synthesis and had a role in ECM formation, the regulation of cell adhesion, and in migration thus suggesting that ASA treatments could benefit osteogenesis differentiation. This was apparent according to the functional enrichment studies (DAVID and PANTHER).

ECM-adhesion proteins were also substantially increased in PDLSCs in the osteogenic medium upon ASA treatments at 1000 μM compared to control. This study demonstrates the ASA might have beneficial effects for osteoblast formation in the presence of agent inducers in osteogenic culture mediums such as ascorbic acid, β-glycerophosphate, and dexamethasone (dex) that play a role in enhancing depositions in the mineralization/ECM.

The ascorbic acid increases collagen biosynthesis [22] while β-glycerophosphate could increase the phosphate ion to produce hydroxylapatite minerals to regulate the expression of osteogenic genes [23]. In addition, dex is capable of promoting osteogenic differentiation by inducing the expression of osteoblast-specific transcription factors such as core-binding factor 1 (cbfa1), and osterix [24].

The other study demonstrated that lower ASA treatments (1 µM and 10 µM) stimulated ST2 cell osteogenic differentiation when compared to higher concentrations (100 and 1000 µM) in the ASA treatment and control groups [25]. An increment of ALP activity and runx2 levels was observed when treated for 7 days. However, in this study, the expression of the osteoblast transcription factor (i.e., Runx2, osterix) was not detected. This is most likely due to the effect of ASA in the osteoblast differentiation.

### ASA treatment and enrichment of canonical pathway

Canonical pathway analysis identified the pathways from the IPA library of canonical pathways that were most significant to the genes expressed differentially in the study. The most significant canonical pathway for all ASA treatments (200, 500, and 1000 μM) was ranked by significance of the *z*-score (Fig. 2). Based on the results, TREM1 signaling was estimated by IPA to be most significantly inhibited affected canonical pathways at 200, 500, and 1000 μM of ASA treatments. However, URA analysis demonstrated that TREM1 was shown positive *z*-score for 500 μM (*z*-score: 2.524) and 1000 μM (z-score: 2.524) of ASA treatments.

### Upstream regulator analysis

URA determine upstream regulator that are connected to dataset genes through set of direct and indirect relationship. It is only based on the prior knowledge of expected effects between TRs and their target genes stored in Ingenuity^®^ Knowledge Base. The activation *z*-score is used to predict upstream regulators based on significant pattern match of up/down regulation. This is the possibility that TREM1 was shown positive z-score in URA analysis but was shown contradict finding (z-score: negative) for the canonical pathway identified in ASA treatments (200, 500, and 1000 μM).

TREM1 proteins are a family of cell surface receptors that participate in diverse cell processes, including inflammation, bone homeostasis, neurological development, and coagulation. TREM1, or the triggering receptor expressed on myleoid cells, is a 30-Kda glycoprotein of the immunoglobulin superfamily that plays an important role in the inflammation process [26]. TREM1 is expressed on blood neutrophils and a subset of monocytes, and is upregulated by bacterial LPS as well as being associated with DAP12 for signaling and function purposes [27]. TREM1 triggered secretion other pro-inflammatory factors including TNF-α, IL-6, CXCL8, CCL4, and CCL5 in order to amplify an inflammation [28].

A direct role of TREM1 signaling in osteoblast differentiation has not been previously observed although their important roles in acute inflammation have been described [22]. The mechanism of TREM1 activation is associated with DAP12 signaling leading to the activation of protein tyrosine kinase and further poshphorylate of various proteins, CA^2T^ mobilization, acivation of extracellular signal-regulated kinases (ERK), and transcription complexes downstream of ERK [27]. TREM1 also induces a pro-inflammatory cytokines factor, TNF-α, and IL-1α, demonstrating that it can amplify pro-inflammatory responses in the presence of induced Toll-like receptors (TLR).

A previous study reports that the co-operation of TREM1 and TLR could produce an inflammatory response. It was observed in this study that ASA treatments at 500 and 1000 μM could block TLR4 activation and further inhibit the inflammatory process. The blocking of TREM1 reduces inflammation and increases survival in animal models of bacterial infections that cause systemic hyperinflammatory syndromes [29].

The shared canonical signaling pathway related to inflammation, which was shown to be inhibited at 500 and 1000 μM of ASA treatments was NF-κB signaling. The NF-κB signaling was activated by pro-inflammatory cytokines such as the tumor necrosis factor (TNF), interleukin1 (IL1), and RANK-L [30]. The TNF was reported to inhibit osteoblast differentiation and bone formation [31] by downregulating the transcription of runx2, which regulates the expression of bone matrix proteins [32].

NF-κB is a transcription nuclear factor that plays a role in the expression and regulator of other pro-inflammatory genes and host immune responses. NFκB signaling also leads to the induction of osteoclast differentiation genes, prolonged survival of osteoclasts, and increased bone resorption. Studies have reported that NF-κB inhibits the osteogenic differentiation of MSCs [33]. In the present study, IPA was predicted upon ASA treatments at 500 and 1000 μM and pointed to the inhibition of several genes including BMP4 and IL1R that cause the suppression of IκB kinase.

The results are in agreement with other studies which reported that the pro-inflammatory genes were stimulated by IKK-NF-κB and impaired the osteogenic differentiation of MSCs [33]. They reported that TNF and IL-17 stimulated IKK- NF-κB and impaired the osteogenic differentiation of MSCs [33]. The inhibition of IKK- NF-κB enhanced the mediation of bone formation. Further evidence in support of NF-κB acting as an important target for bone disease and tissue regeneration is provided by Chen et al. who report that DNA damage could inhibit osteogenic differentiation of MSC and accelerate bone aging by activating NF-κB in vitro and in vivo [34].

Renin-angiotensin signaling (RAS) was inhibited at the highest concentrations of ASA treatments (1000 μM). RAS plays an important role in regulating of cardiovascular and renal functions to maintain extracellular fluid volumes and electrolyte homeostasis [31, 35]. In addition, RAS in bones remodeling has also been noted to negatively regulate bone turnover and bone mass via osteoclast AT1 receptors [36].

Nakai et al. also report that Ang II suppressed ALP activity on osteoblastic cells and ROS17/2.8 during the growth stage [37]. They further investigated the effect of Ang II on the expression of the transcription factor of osteoblast runx2 and found that Ang II suppresses osteoblastic differentiation by decreasing runx2 and Msx2 expression and suppresses mineralized nodule formation in ROS17/2.8 cells.

Interestingly, cyclin-dependent kinase 5 (CDK5) signalings were inhibited at 500 μM of ASA treatments only. CDK5 is expressed proline-directed serine/threonine kinase [38]. The best-delineated role of CDK5 is its regulation of the cytoskeleton architecture of the central nervous system (CNS) during inflammatory hyperalgesia. There are not many studies that provide a description or function of CDK5 in dental stem cell differentiation.

However, CDK5 has been seen to be expressed in odontoblast-like cells and odontoblast-enriched primary preparations from murine teeth and an odontoblast-like cell line (MDPC-23) [39]. They found that CDK5 is functionally active in these cells and its kinase activity is upregulated during cell differentiation in MDPC-23 cells. They observed that CDK5 and p35 are expressed in a murine odontoblast-enriched primary preparation of cells from teeth. CDK5 is also functionally active in odontoblast-like MDPC-23 cells.

Their findings are based on the theory that odontoblasts are directly involved in dental nociception and pain transduction [39]. This study shows that the ASA treatments were downregulated and genes laminin were bound tightly to integrin alpha 6 beta 1 (Igα6) and further activated the P35 signaling via ERK1/2. This indicates that ASA could be a novel therapeutic agent for use in periodontal regeneration/osteoblast differentiation.

Meanwhile the gene INHBβ was found to be highly expressed in PDLSCs proliferation with FC (+3.25±0.32) but showed to contradict with PDLSCs in osteogenic culture media which were downregulated at 1000 μM of ASA treatment with FC (−3.2219) but with no significant changes at 200 and 500 μM of ASA treatments. The difference of expression in proliferation and differentiation of certain genes is probably due to the different mediums used in the experiment. ASA was treated in the normal media to study the effects of proliferation of PDLCs and PCR assays while the next study used osteogenic media containing osteogenic inducers such as ascorbic acid, dexametahasone, and β-glycerophosphate in the microarray studies. This indicated that the type of media could alter the effect of ASA in modulating PDLSCs gene expression profile.

### Validation of microarray data by qPCR

Seven genes were validated by qPCR namely Fibronectin1 (FN1), Integrin Alpha 5 (Igα5), Bone Morphogenetic Protein Endothelial Cell Precursor-derived Regulator (BMPER), Bone Morphogenic Protein4 (BMP4), Fibroblast Growth Factor1 (FGF1), Fibroblast Growth Factor5 (FGF5), and Fibroblast Growth Factor Receptor1 (FGFRL1). Seven genes selected for validation were associated with osteogenesis differentiation. Our observations hypothesized that ASA promotes osteogenesis by targeting the FGF/FGFRL pathway and modulates fibronectin and integrin interaction. ‘The study showed FGF1 and FGFRL1 to be highly expressed from the highest ASA treatments at FC 2.1459 and 2.1035 respectively. The upregulation of FGFRL1 and FGF1 was also reported in human dental follicles in response to DMEM media and dexamethasone suggesting that FGF2, FGF1, FGF3, and FGFRL1 play a critical role in the differentiation of dental follicle cells [40].

Based on this study it has been suggested that ASA acts as a regulator for FGF1 to bind to the FGFRL1 receptor and activates several bone-related marker genes. FGFs are heparin binding proteins and signal through a binding to the tyrosine kinase in the intracellular region of the FGR receptor (FGFR). The FGFRs contain an extracellular ligand-binding domain, a transmembrane region, and an intracellular divided tyrosine kinase domain. The binding of FGFs to FGFRs enables the autophosphorlyation of tyrosine in the intracellular region of FGFR leading to the activation of intracellular downstream signaling pathways, such as mitogen-activated protein kinase (Ras/MAPK), protein kinase B, protein kinase C, and phospholipase C and also the p21 pathways [41].

On the other hand, Miraoui et al. reported that FGFR2 acts as novel regulatory molecules that promote osteogenic differentiation in murine MSCs [42]. The effect of FGFR2 is mediated by PKCα and ERK1/2 pathways that have critical roles in FGFR2-induced osteogenic differentiation of murine MSCs [42].

The present study also found that the osteoblast differentiation mechanism not only depended on FGFs but also on the BMPs gene. In this study, BMP1 was observed to downregulate upon all ASA treatments while BMP4 was downregulated at high concentration of ASA treatments. BMP1 was also differentially expressed in dental follicle stem cells. BMP1 is not part of the TGF-b family and, as a procollagen C proteinase containing 730 amino acid residues with rich cysteine residue, it is a regulatory factor for bone growth and belongs to the family of metalloproteinases [43].

The expression of BMPER was noted at 1000 μM of ASA treatments. The present study believe that BMPER acts as the mode and action and is a novel regulator in osteoblast-like differentiations as previously reported [44]. BMPER is a differentially expressed protein in embryonic endothelial precursor cells. MSCs reside in a perivascular niche of the body, suggesting that they interact closely with vascular endothelial cells through cell–cell interactions or paracrine signaling to maintain cell functions.

The anatomical relationship between human MSCs and vascular endothelial cells also suggests that these two cell types interact with each other most likely through cell–cell interaction and/or paracrine signaling. Endothelial cells can regulate the cellular activities of human MSCs and have demonstrated the ability to enhance osteogenic differentiation of human MSCs through direct cell–cell contact [45]. BMPER encodes protein that interacts and inhibits BMPs function. It interacts with BMP2, BMP4, and BMP6 and antagonized BMP4-dependent SMAD5 activation [46, 47]. It has been shown to inhibit BMP2 and BMP4-dependent osteoblast differentiation and BMP-dependent differentiation of the chondrogenic cells. This seems to tally with this present study when BMP4 was downregulated in this study upon ASA treatments.

## Conclusions

ASA may potentially enhance periodontal regenerative processes through the stimulation of selected number of growth factors-associated genes in PDLSCs or/and via its enhancement of osteogenic potential–these observations suggest ASA could be supportive of regenerative processes and may help the improvement in periodontal health. This study showed ASA was capable of enhancing the proliferation and osteogenic differentiation of PDLSCs grown in osteogenic media.

This study postulated that ASA promotes osteogenesis by targeting the FGF/FGFRL pathway and modulates fibronectin and integrin interaction. Further in-depth investigations, such as global proteome and transcriptome profiling studies may provide additional insights on the impact of ASA on PDLSCs regenerative activities and how it could affect PDL functions in periodontal health and regeneration.
